# Beyond Antibiotics: Traditional Chinese Medicine and Flavonoids in the Management of Endometritis

**DOI:** 10.3390/vetsci13070635

**Published:** 2026-06-30

**Authors:** Abdul Qadeer, Mohamed Tharwat, Ibrahim F. Halawani, Fuad M. Alzahrani, Khalid J. Alzahrani, Fahad A. Alshanbari, Muhammad Zahoor Khan

**Affiliations:** 1 Department of Medical Sciences, Shandong Xiehe University, Jinan 250109, China; qadeerabdul@sdxiehe.edu.cn; 2 Department of Clinical Sciences, College of Veterinary Medicine, Qassim University, P.O. Box 6622, Buraidah 51452, Saudi Arabia; atieh@qu.edu.sa; 3 Department of Clinical Laboratory Sciences, College of Applied Medical Sciences, Taif University, Taif 21974, Saudi Arabia; 4 Department of Medical Biosciences, College of Veterinary Medicine, Qassim University, P.O. Box 6622, Buraidah 51452, Saudi Arabia; 5 College of Agriculture and Biology, Liaocheng University, Liaocheng 252000, China

**Keywords:** endometritis, chronic endometritis, flavonoids, traditional Chinese medicine, NF-κB, NLRP3 inflammasome, Nrf2, ferroptosis, gut–uterus axis, antimicrobial resistance

## Abstract

Endometritis—inflammation of the endometrial lining—imposes a substantial reproductive and economic burden in both human gynecology and livestock production, where it is a leading cause of recurrent implantation failure in humans and the costliest reproductive disorder in cattle. Conventional management is overwhelmingly antibiotic-based, yet escalating antimicrobial resistance, tissue and milk residues, microbiota disruption and high relapse rates have eroded its efficacy and acceptability, creating an urgent need for mechanism-based, host-directed alternatives. Here we synthesize the expanding evidence positioning dietary flavonoids and traditional Chinese medicine (TCM) formulations as such interventions. Across diverse compounds and preparations, anti-endometriotic activity converges on a tractable set of molecular nodes: TLR4/NF-κB signaling, the NLRP3 inflammasome–pyroptosis axis, the Keap1/Nrf2/HO-1 antioxidant program, PI3K/AKT and PPAR-γ signaling, ferroptosis, and the gut–uterus microbial–metabolite axis. Veterinary field studies report cure rates and fertility outcomes rivaling first-line antibiotics, while integrative case reports show benefit in antibiotic-refractory human chronic endometritis. Translation remains constrained by poor bioavailability, formulation heterogeneity, over-reliance on lipopolysaccharide-only models and a scarcity of randomized trials—barriers now addressable through nanocarrier delivery, network-pharmacology-guided standardization and biomarker-stratified designs. Flavonoids and TCM are best viewed not as substitutes for antibiotics but as a mechanistically rational, multi-target strategy aligned with One Health antimicrobial stewardship.

## Simple Summary

Endometritis, an inflammation of the lining of the uterus (the endometrium), is a common and costly problem affecting both women and farm animals, where it frequently causes infertility, repeated pregnancy loss, and heavy economic losses in dairy cattle. Doctors and veterinarians usually treat it with antibiotics, but these drugs are becoming less effective as bacteria grow resistant, and they can leave harmful residues and disturb the body’s natural balance of microbes. This review explores a promising alternative: natural plant compounds called flavonoids and traditional Chinese medicine formulations. We bring together what laboratory studies, animal trials, and early human reports reveal about how these natural agents calm harmful inflammation, protect tissue, and restore a healthy microbial community. Rather than replacing antibiotics, we argue that they work best alongside them, reducing antibiotic use and improving outcomes. Our aim is to highlight their potential and outline the practical steps still needed for everyday use.

## 1. Introduction

Endometritis is a clinically and etiologically heterogeneous inflammation of the endometrial lining that spans an acute, often overtly purulent disease typical of the early postpartum period in livestock [1,2,3], and of post-partum or post-abortive sepsis in women, and a low-grade, frequently asymptomatic chronic form (CE) increasingly recognized as a correctable cause of subfertility and recurrent pregnancy loss [4,5]. In assisted reproduction, CE has been identified in 30–66% of women with recurrent implantation failure (RIF) and in 12–46% of those with recurrent pregnancy loss, and its correction substantially improves embryo transfer outcomes [6,7,8]. In dairy systems, clinical and subclinical postpartum endometritis affects 15–40% of cows and is the largest single driver of extended calving intervals, culling and lost milk yield, generating losses estimated at >€1.4 billion annually in the European Union alone [9,10,11,12,13].

Mechanistic dissection over the past decade has revised the classical view of endometritis as a purely infectious disorder amenable to bacterial eradication. While ascending Gram-negative organisms—principally *Escherichia coli*, *Trueperella pyogenes*, *Fusobacterium necrophorum* and *Prevotella* spp. in livestock [13,14,15], and a more diverse polymicrobial signature dominated by *Streptococcus*, *Enterococcus*, *Enterobacteriaceae* and intracellular organisms in humans [16]—are necessary triggers, the magnitude and persistence of endometrial injury are determined by the host innate-immune response. Bacterial lipopolysaccharide engagement of TLR4 on endometrial epithelial cells, stromal fibroblasts and resident macrophages activates MyD88-, TRAF6- and TAK1-dependent NF-κB and MAPK signaling, drives transcription of TNF-α, IL-1β, IL-6, IL-8 and the chemokines that recruit polymorphonuclear neutrophils, and primes the NLRP3 inflammasome for caspase-1–dependent maturation of IL-1β and IL-18 and for gasdermin D-mediated pyroptosis [17,18,19]. The resulting cytokine and reactive-oxygen-species burden disrupts the tight-junction proteins ZO-1, occludin and claudins, perpetuating barrier breakdown, propagating ferroptotic and apoptotic cell death, and impairing the decidualization and receptivity programs required for successful implantation [20,21,22]. A parallel disruption of the cervicovaginal–uterine microbial continuum—with depletion of Lactobacillus and expansion of Proteobacteria—reinforces this maladaptive inflammatory state and is increasingly viewed as both consequence and cause of chronic disease [23,24].

This expanded pathophysiological framework reveals two converging therapeutic imperatives that pure antibacterial chemotherapy cannot satisfy: (i) the need to attenuate dysregulated host inflammation, oxidative stress, and regulated cell death independently of bacterial burden, and (ii) the need to restore, rather than further perturb, the mucosal microbial ecosystem. It is against this backdrop that plant-derived polyphenols—particularly flavonoids—and the multi-component formulations of traditional Chinese medicine (TCM) have re-emerged as compelling candidates. Flavonoids modulate, rather than simply suppress, the TLR4/NF-κB, NLRP3, Keap1/Nrf2/HO-1, PI3K/AKT, PPAR-γ and ferroptosis pathways that orchestrate endometrial inflammation, and several—most strikingly hyperoside—now have a demonstrated capacity to remodel the gut–uterus axis through specific microbial metabolites [23]. TCM formulations, refined empirically over centuries for the indication ben qi—broadly equivalent to postpartum reproductive-tract inflammation—are increasingly being deconvoluted by network pharmacology and multi-omics into mechanistically coherent multi-target interventions [25,26].

In this review, we systematically integrate the molecular, preclinical, and emerging clinical evidence supporting flavonoids and TCM as next-generation, host-directed interventions for endometritis. We first situate this evidence within the current standard of care, summarizing where antibiotic monotherapy succeeds, where it fails, and why an adjunctive or alternative pharmacology is now required. We then dissect the convergent mechanistic nodes—TLR4/NF-κB, NLRP3/pyroptosis, Nrf2-governed redox defense, PI3K/AKT and PPAR-γ signaling, ferroptosis and microbiota remodeling—through which individual flavonoids and TCM preparations act, and synthesize the translational evidence from rodent models, naturally affected livestock and the small but growing human case series. Finally, we critically appraise the principal barriers to clinical translation—pharmacokinetics, formulation heterogeneity, model relevance and trial design—and propose a tractable roadmap for moving the field from mechanistic plausibility to clinical impact.

## 2. Literature Search Methodology

This narrative review draws on literature identified through a structured, though non-systematic, search of PubMed, Web of Science, Scopus and Google Scholar, covering the period from database inception through June 2026. Search strategies combined terms for endometritis and related uterine inflammation with three thematic clusters: (i) bioactive compounds, including flavonoids and polyphenols; (ii) traditional Chinese medicine and herbal medicine; and (iii) key mechanistic concepts, namely NF-κB, the NLRP3 inflammasome, Nrf2 signaling, ferroptosis, the gut–uterus axis, and antimicrobial resistance. The reference lists of included articles were further screened to capture relevant sources not retrieved in the initial search.

Studies were selected for discussion where they reported molecular mechanisms, preclinical efficacy, or clinical and veterinary applications of flavonoids or traditional Chinese medicine in endometritis and closely related conditions. Non-English and non-Chinese publications, abstract-only conference items, and studies addressing endometriosis alone were not considered. Consistent with the aims of a narrative review, this approach prioritized conceptual and mechanistic relevance over exhaustive coverage, allowing the synthesis to focus on the most informative and representative work in the field.

To situate this synthesis within the broader research landscape, a brief survey of the Web of Science Core Collection (2010–June 2026) was also undertaken. This survey indicates a field that has expanded rapidly since 2020, with the majority of records published within the last four years. Keyword co-occurrence patterns point to NF-κB, the NLRP3 inflammasome, oxidative stress and Nrf2 signaling, gut microbiota, and ferroptosis as the dominant research themes, closely mirroring the mechanistic framework adopted in this review. The gut–uterus axis and ferroptosis, in particular, have emerged as distinct themes only since 2023, marking them as the newest frontiers of the field and underscoring the timeliness of the present review.

## 3. Flavonoids and Traditional Chinese Medicine as Antibiotic Resistance-Breakers, Not Replacements, in Endometritis: Rationale for Adjunctive Therapy

Antimicrobial resistance (AMR) is a present clinical emergency [27]. The GRAM study attributed approximately 1.27 million deaths directly to bacterial AMR in 2019, with a further 4.95 million associated—surpassing HIV/AIDS and malaria [28,29,30]. Projections are more sobering: over 39 million AMR-attributable deaths are expected between 2025 and 2050, with annual direct mortality approaching 1.9 million by mid-century [31]. Sustained antimicrobial overuse across human medicine, animal agriculture [32,33,34] and aquaculture maintains the selective pressure perpetuating this crisis [35].

The rationale for exploring natural products—particularly flavonoids and TCM formulations—as adjunctive agents in this context rests on three converging lines of evidence. Flavonoids possess intrinsic multi-target pharmacology: unlike single-mechanism antibiotics, they concurrently modulate host inflammatory signaling (NF-κB, NLRP3), oxidative stress (Nrf2/HO-1), regulated cell death (ferroptosis, pyroptosis), and mucosal microbiota composition, thereby addressing the host-driven pathology that persists independently of bacterial burden. Several flavonoids and TCM preparations have demonstrated direct antibiofilm, anti-quorum-sensing, and efflux-pump-inhibitory activities that restore antibiotic susceptibility in resistant organisms, functioning as genuine resistance-breakers rather than antimicrobial replacements. TCM formulations have been empirically refined over centuries for indications functionally equivalent to postpartum uterine inflammation, and modern network pharmacology is now validating these as mechanistically coherent multi-component interventions whose efficacy arises from the synergistic engagement of complementary molecular targets. Collectively, these properties position flavonoids and TCM not as alternatives to antibiotics but as mechanistically rational adjuncts that fill the therapeutic void created by resistance, biofilm tolerance, and dysbiosis.

Endometritis holds a disproportionate position within this picture [36]. Key resistant pathogens include *Enterococcus faecalis*, *Streptococcus agalactiae*, *E. coli*, *Klebsiella pneumoniae*, *Chlamydia trachomatis* and *Mycoplasma genitalium* in endometritis [16,37,38,39]. ESBL-producing *E. coli* and *K. pneumoniae* negate cephalosporins [40], while fluoroquinolone-resistant Enterobacteriaceae compress salvage options [38,39,41]. *M. genitalium* has undergone one of the most rapid resistance trajectories documented for any pathogen: macrolide resistance via 23S rRNA mutations predominates in several high-prevalence settings, while concurrent fluoroquinolone resistance is emerging globally, yielding multidrug-resistant phenotypes refractory to both available drug classes [42,43,44].

Critically, acquired resistance explains only part of antibiotic failure in endometritis; four additional biological mechanisms undermine efficacy independently of acquired resistance within the microbial community. First, *T. pyogenes*, *E. coli*, and *F. necrophorum* form polymicrobial biofilms on the denuded endometrial surface, elevating effective tolerance by one to three orders of magnitude through restricted drug diffusion, metabolic quiescence, and efflux pump induction [45,46,47]. Within these multispecies communities, interspecies coexistence restructures biofilm architecture and extracellular matrix composition, amplifying antimicrobial recalcitrance beyond levels attributable to any single constituent organism [48,49]; efflux activity and biofilm-mediated tolerance are synergistically coupled, with pump induction establishing local antibiotic concentration gradients that further reduce effective intrabiofilm drug exposure [50]. The pathogenic contribution of *T. pyogenes* to these community-level dynamics has been confirmed in bovine endometritis, where elevated minimum inhibitory concentrations and virulence-associated genotypes independently compound treatment resistance [51,52]. Second, *E. coli*, *T. pyogenes*, *C. trachomatis*, and *M. genitalium* persist intracellularly within endometrial epithelial cells and macrophages, exploiting a pharmacological sanctuary inaccessible to β-lactams and aminoglycosides: *T. pyogenes* has been shown to survive within host phagocytes for up to 72 h [53], *C. trachomatis* converts to non-replicating but metabolically viable aberrant bodies under immune pressure, recovering infectivity upon nutrient restoration [54], and *E. coli* subpopulations survive high-dose β-lactam exposure through phenotypic persistence mechanisms entirely independent of acquired resistance determinants [55]; this pattern is consistent with the broader, cross-species phenomenon of intracellular bacterial persisters as pharmacological sanctuaries driving chronic treatment failure [56]. Third, the syntrophic relationship between aerobic *E. coli* and obligate anaerobes generates composite community-level resistance exceeding the sum of individual constituents—a phenomenon invisible to single-organism susceptibility testing [57,58,59]; this fundamental limitation of conventional diagnostic methodology has direct clinical consequences, as demonstrated by a prospective 2020–2024 cross-sectional study documenting 98.5% ampicillin resistance and 34.7% extended-spectrum β-lactamase positivity among endometritis pathogens despite empirical first-line therapy guided by standard susceptibility profiles [39], while the synergistic interplay between facultative aerobes and obligate anaerobes in polymicrobial infections consistently generates resistance phenotypes that cannot be predicted from, or detected by, individual isolate susceptibility data alone [60,61,62,63]. Fourth, and counterintuitively, broad-spectrum regimens themselves perpetuate disease by depleting *Lactobacillus* dominance, elevating vaginal pH, and facilitating colonization by pathobionts [23,24]; collateral gut dysbiosis further impairs uterine immune tone via the gut–uterus axis [23]. In veterinary practice, these failure modes are compounded by EU Regulation 2019/6 and analogous restrictions withdrawing critically important antimicrobials precisely as resistance to alternatives intensifies.

Taken together, these dimensions—acquired resistance, biofilm-mediated tolerance, intracellular persistence, polymicrobial composite resistance, antibiotic-driven dysbiosis, and tightening regulation—define a therapeutic void that is mechanistic, not merely pharmacological, and cannot be closed through dose optimization or novel combination regimens alone. What is required are adjunctive interventions that target host inflammatory signaling rather than bacterial metabolism alone, that restore rather than disrupt the mucosal microbial ecosystem, and that do not contribute to the global resistome. Flavonoids and TCM formulations are therefore most appropriately positioned as resistance-breakers—agents that restore antibiotic susceptibility, disrupt biofilms, and attenuate endometrial inflammation—rather than as replacements for antimicrobial therapy. The molecular and clinical evidence supporting this role is examined in Section 4 and Section 5.

## 4. Flavonoids in the Management of Endometritis

### 4.1. Rationale for a Flavonoid-Based Strategy

Endometritis—inflammation of the endometrial lining—constitutes a major reproductive and economic burden in both women and livestock, and its conventional management relies heavily on antibiotics. This dependence is increasingly untenable owing to antimicrobial resistance, tissue drug residues and an associated risk of relapse, motivating the search for safe, multi-target alternatives [25,64]. Oxidative stress and dysregulated innate-immune signaling are now recognized as central drivers of disease progression, and the closely related disorder endometriosis affects approximately 10% of reproductive-aged women worldwide with no definitive cure [65]. Flavonoids—a structurally diverse class of plant polyphenols abundant in fruits, vegetables, and traditional medicinal herbs—have consequently emerged as promising nutraceutical candidates, since they concurrently temper inflammation, restore redox balance, and remodel the host microbiota through several convergent molecular pathways [65]. This section synthesizes the preclinical and clinical evidence for individual flavonoids and flavonoid-rich preparations, organized by their dominant mechanisms of action.

### 4.2. Suppression of TLR4/NF-κB-Driven Inflammation

The Toll-like receptor 4 (TLR4)/nuclear factor-κB (NF-κB) cascade is the most frequently reported target in flavonoid studies, reflecting its pivotal role in transducing signals from bacterial endotoxin into the release of pro-inflammatory cytokines. Engeletin (dihydrokaempferol 3-rhamnoside), a flavanonol glycoside of white grapes, dose-dependently attenuated uterine injury and myeloperoxidase (MPO) activity in lipopolysaccharide (LPS)-induced murine endometritis by inhibiting TLR4 together with the downstream adapters MyD88, IRAK1, TRAF6 and TAK1, suppressing iNOS and COX-2, and blocking NF-κB-p65 nuclear translocation [19]. Astragalin (kaempferol 3-glucoside) acted comparably, curbing TNF-α, IL-1β and IL-6 and the phosphorylation of p38, p65, ERK and JNK in *Leptospira*-infected uterine cells [66], and suppressing the TLR4/NF-κB axis in *E. coli*-challenged sheep endometrial epithelial cells [67], consistent with its broader modulation of NF-κB, MAPK and JAK/STAT networks [68,69]. Icariin, a prenylated flavonol from *Epimedium brevicornum*, similarly repressed TLR4-associated NF-κB activation while elevating anti-inflammatory IL-10, and concurrently engaged the antioxidant Nrf2 axis described below [70].

**Figure 1:**
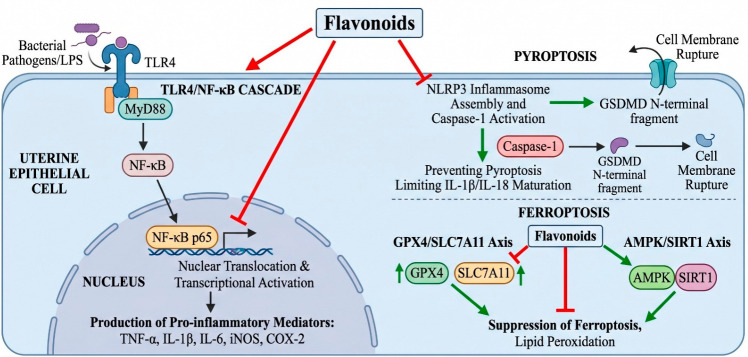
Flavonoids suppress inflammatory signaling in endometritis via two main pathways.

Whole-plant and total-flavonoid preparations recapitulate this mechanism. The active constituents of *Syringa oblata* Lindl. (SOL)—predicted by network pharmacology to be luteolin, kaempferol, oleanolic acid and rutin—ameliorated *Staphylococcus aureus*-induced uterine injury and bacterial load via the TLRs/NF-κB pathway, and a green deep-eutectic-solvent ultrasound-assisted extraction (DES-UAE) was optimized to enrich these flavonoids [64]. The anti-endometriotic activity of *Urtica dioica* L. was likewise attributed to its flavonoid constituents—rutin, isoquercetin, nicotiflorin, narcissin, and astragalin—which reduced peritoneal TNF-α, VEGF, and IL-6 and reduced implant volume in a rat model [71] (Figure 1).

In uterine epithelial cells challenged by bacterial pathogens or LPS, flavonoids act on two convergent axes. First, they block the TLR4/MyD88/NF-κB cascade, reducing nuclear translocation of NF-κB p65 and downstream production of TNF-α, IL-1β, IL-6, iNOS and COX-2. Second, they inhibit NLRP3 inflammasome assembly and caspase-1 activation, thereby preventing pyroptosis and limiting IL-1β/IL-18 maturation, while additionally suppressing ferroptosis via the AMPK/SIRT1 and GPX4/SLC7A11 axes. Together, these actions attenuate uterine tissue injury. Red blunt arrows show inhibition; green arrows represent activation/restoration.

### 4.3. Inhibition of NLRP3 Inflammasome Activation and Pyroptosis

Several flavonoids extend their anti-inflammatory effects to the NLRP3 inflammasome, whose assembly drives caspase-1-dependent pyroptosis. Total flavonoids of *Clinopodium chinense* (TFC), a centuries-old remedy for gynecological hemorrhage, suppressed the TLR4/NF-κB/NLRP3 axis in vivo and in primary mouse endometrial epithelial cells (MEECs); UPLC-Q-TOF-MS identified six plasma-absorbed constituents, and TFC reduced MPO activity, IL-18, IL-1β and TNF-α and inhibited caspase-1, ASC, NLRP3 and GSDMD, thereby preventing pyroptosis [18]. Epigallocatechin-3-gallate (EGCG) conferred similar protection by upregulating SIRT1 and downregulating NLRP3, ASC, and caspase-1, thereby reducing oxidative stress and apoptosis in murine uteri and bovine endometrial epithelial cells [72]. Puerarin engaged the P2X7 receptor/NLRP3 axis to limit *S. aureus*-induced inflammation while restoring GPX4 and SLC7A11 to suppress ferroptosis [73], an action mechanistically linked to AMPK/SIRT1 signaling in LPS models, since the AMPK inhibitor compound C and the SIRT1 inhibitor EX-527 reversed its protective effects [74] (Figure 1).

### 4.4. Restoration of Redox Balance and Inhibition of Ferroptosis

**Figure 2:**
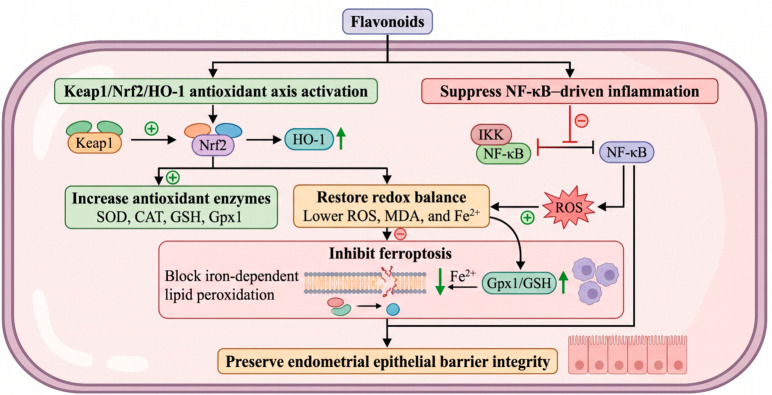
Flavonoids restore redox balance and inhibit ferroptosis in endometritis. By activating the Keap1/Nrf2/HO-1 antioxidant axis and suppressing NF-κB–driven inflammation, flavonoids lower ROS, MDA, and Fe^2+^ while raising antioxidant enzymes (SOD, CAT, GSH, Gpx1). These effects block iron-dependent lipid peroxidation (ferroptosis) and preserve endometrial epithelial barrier integrity.

A complementary theme is the activation of the Kelch-like ECH-associated protein 1 (Keap1)/nuclear factor erythroid 2-related factor 2 (Nrf2)/heme oxygenase-1 (HO-1) antioxidant program (Figure 2). Liquiritin, derived from *Glycyrrhiza glabra*, inhibited apoptosis and activated Keap1/Nrf2/HO-1 in LPS-stimulated human endometrial epithelial cells, lowering ROS while increasing superoxide dismutase and catalase; pathway dependence was confirmed because the Nrf2 inhibitor ML385 abolished its effects [75]. Apigenin operated through a parallel NF-κB/Nrf2/HO-1 mechanism, suppressing MPO and pro-inflammatory cytokines while elevating Nrf2 and HO-1 [76]. Fisetin combined TLR4–ROS/NF-κB inhibition with Nrf2/HO-1 activation in both murine and bovine models, and Nrf2 silencing abrogated its protection [77]; in a rat endometriosis model, it further reduced lesion size, mast-cell-derived NLRP3 signaling, TGF-β and α-SMA [78].

Ferroptosis—iron-dependent, lipid-peroxidative cell death—has emerged as a unifying downstream event. Luteolin, a dietary flavone with broad activity across NF-κB/MAPK, PI3K/AKT/PTEN, TGF-β/Smad and steroid-receptor signaling [79], inhibited both inflammation and ferroptosis in *S. aureus*-induced endometritis: it lowered MDA and Fe^2+^, raised GSH, restored ZO-1 and occludin, and acted through Nrf2, with protection lost in Nrf2-knockdown mice [21]. In LPS models, luteolin again modulated TLR4-associated Nrf2 and NF-κB signaling, scavenging MDA and ROS while boosting SOD1, CAT and Gpx1 [22]. Beyond its signaling effects, luteolin displayed direct antibiofilm activity against *Trueperella pyogenes* (MBIC 156 µg/mL; MBEC 312 µg/mL), dispersing biofilms and downregulating *luxS*, *plo*, *rbsB*, and *lsrB*, with a corresponding relief of experimental endometritis [80]. Morin [81] and hesperidin likewise targeted ferroptosis and mitochondrial oxidative stress, the latter through direct AMPK binding and AMPK/PGC-1α activation confirmed by molecular docking, CETSA and SPR [20].

### 4.5. Modulation of Pi3k/akt, Ppar-Γ, and Fibrotic Signaling

A further mechanistic cluster involves the phosphoinositide 3-kinase (PI3K)/AKT and peroxisome proliferator-activated receptor-γ (PPAR-γ) pathways. Safflower total flavonoids (STF) from *Carthamus tinctorius* alleviated LPS injury in human Ishikawa cells by activating ERα/PI3K/AKT while suppressing ASK1 and caspase-3/-11 [82]; in an incomplete-abortion rat model, STF repaired uterine damage, shifted the gut microbiota toward normal and acted through ERα/PI3K/AKT and MAPK networks, with network pharmacology implicating TNF, IL6, TP53, AKT1, JUN, VEGFA and CASP3 as core targets [83]. Naringin took the opposite directional approach, inhibiting the endoplasmic reticulum stress (ERS)–PI3K/AKT axis to reduce unfolded-protein-response-driven autophagy and apoptosis [84]. Astilbin from *Smilax china* acted in a PPAR-γ-dependent manner, interrupting the positive feedback between NF-κB and JAK2/STAT3 signaling—an effect abolished by PPAR-γ siRNA or antagonist and corroborated by molecular docking [85], while cyanidin-3-O-glucoside coupled NF-κB suppression with PPARγ/ABCA1 activation, its protection reversed by the PPARγ inhibitor GW9662 [86]. Hyperoside also repressed the pro-fibrotic TGF-β/Smad3 pathway, attenuating endometrial fibrosis and inflammation in an intrauterine adhesion rat model [87].

### 4.6. The Gut–Uterus Axis and Microbiota Remodeling

An emerging, mechanistically distinct paradigm is the modulation of disease through the gut microbiota. Hyperoside, a flavonol glycoside abundant in hawthorn, alleviated *E. coli*-induced murine endometritis (80 mg kg^−1^) by enriching beneficial *Lactobacillus* and *Prevotella*, which elevated the microbial metabolite hydroxyphenyllactic acid (HPLA); circulating HPLA reached the uterus, bound TLR4 and suppressed TLR4/NF-κB signaling, lowering TNF-α, IL-1β, IL-6 and IL-18 [23]. The causal role of microbiota restructuring was confirmed by antibiotic depletion and fecal microbiota transplantation, positioning hyperoside as a prototype gut–uterus-axis therapeutic. Consistent with this concept, STF also normalized the gut microbiota of treated rats as part of its multi-target action [82].

### 4.7. Whole-Plant Preparations and Translational Veterinary Evidence

The translational case for flavonoids is strengthened by veterinary studies in naturally affected livestock, where antibiotic alternatives are urgently needed. A traditional Chinese medicine formula combining *Viola yedoensis* and *Leonurus japonicus* (TCMF) reduced IL-1β, IL-6, IL-8 and IL-18 in LPS-stimulated cells and lowered uterine index, bacterial load and TNF/PTGS2/CASP3 expression in rats; in dairy cows it cut uterine-discharge scoring, polymorphonuclear-neutrophil (PMN) count and bacterial load, raised albumin, superoxide dismutase and cortisol, and achieved an 80% cure rate via the TNF signaling pathway [25]. Baicalin attenuated clinical, cytological, and histopathological signs in a rabbit model of cow endometritis and suppressed NF-κB and JNK signaling in LPS-treated macrophages [88]. The Micronized Purified Flavonoid Fraction (MPFF), a citrus-derived diosmin/hesperidin preparation, improved cure rate, uterine involution, bacteriology and subsequent fertility in a dose-dependent manner in cows with *E. coli* metritis [89].

Flavonoid-rich botanical extracts have shown comparable promise. *Eucalyptus robusta* leaf extract produced curative and protective effects against bacterial endometritis in rats that matched or exceeded cefixime, reducing TLR-4, TLR-9, COX-2, iNOS, serum amyloid A and MPO [90]; oral *Aegle marmelos* and *Murraya koenigii* leaf powder lowered bacterial load and PMN count while raising endogenous antioxidants in repeat-breeding dairy cows [91]; and a *Garcinia mangostana*–*Achyranthes aspera* gel inhibited endometritis-associated bacteria in cattle without hepatic or renal toxicity [92]. Not all preparations succeeded, however: oral *Echinops spinosus* decoction failed to prevent clinical endometritis in postpartum cows and was associated with poorer reproductive outcomes [93], a negative result that usefully tempers expectations and underscores the importance of compound selection, dose and delivery route.

Beyond the individual preparations discussed above, the broader literature on flavonoids and gut microbiota modulation provides additional mechanistic support for the gut–uterus axis concept in endometritis. Quercetin, one of the most widely studied dietary flavonoids and a recurrent constituent in TCM network pharmacology analyses, has been shown to increase the relative abundance of *Akkermansia muciniphila* and to restore short-chain fatty acid (SCFA) production—particularly butyrate—in models of intestinal dysbiosis; butyrate is a potent inducer of regulatory T cells and an inhibitor of NF-κB in distant mucosal tissues, providing a plausible systemic link between gut remodeling and uterine immune modulation. Similarly, baicalin, the principal flavonoid glycoside in Scutellaria-based formulations, has been reported to suppress Clostridioides and Enterobacteriaceae while enriching *Lactobacillus* spp. in antibiotic-induced dysbiosis models, and to attenuate distant organ inflammation through microbiota-dependent bile acid metabolism. These observations, together with the demonstrated causal role of hyperoside-driven HPLA production and the gut microbiota normalization achieved by safflower total flavonoids and Baogong Decoction, suggest that microbiota remodeling is not an incidental effect of oral flavonoid administration but rather a therapeutically relevant and potentially predictive mechanism of action that warrants systematic evaluation across all candidate compounds.

### 4.8. Translational Outlook

Several important caveats temper the strength of the current evidence and must be acknowledged. First, the overwhelming majority of studies are preclinical, conducted in rodent models with acute LPS-induced endometritis, and the relevance of these findings to chronic, naturally occurring, polymicrobial disease in humans and livestock remains to be established. Second, sample sizes in both in vivo and in vitro studies are generally small (typically *n* = 6–10 per group in animal experiments), limiting statistical power and generalizability. Third, most studies report only positive outcomes, raising concern about publication bias; the inclusion of the negative Echinops spinosus result is notable precisely because it is exceptional. Fourth, the reproducibility of findings across independent laboratories and in different animal strains or cell lines has rarely been tested. Fifth, study designs are heterogeneous with respect to dose, duration, route of administration, and outcome measures, precluding meaningful meta-analysis. These limitations do not negate the mechanistic plausibility of flavonoid-based interventions but do mandate a substantially higher standard of evidence before clinical translation can be recommended.

Collectively, the evidence depicts flavonoids as genuinely multi-target agents that converge on a limited set of nodes—TLR4/NF-κB, the NLRP3 inflammasome, Nrf2-governed redox defense, PI3K/AKT and PPAR-γ signaling, ferroptosis and the gut–uterus axis—offering a mechanistic breadth that single-target antibiotics cannot match. The principal barrier to clinical translation remains pharmacokinetic: luteolin and many congeners suffer from poor aqueous solubility and low oral bioavailability [79]. Emerging nanocarrier and prodrug strategies are beginning to overcome these limitations, and the convergence of robust mechanistic data, encouraging livestock efficacy and an improving delivery toolkit warrants well-designed clinical trials to establish flavonoids as non-antibiotic interventions for endometritis and related inflammatory reproductive disorders [65,79]. A summary of the various flavonoids and their roles in endometritis is presented in Table 1.

## 5. Traditional Chinese Medicine in Endometritis

Before reviewing individual formulations, it is important to clarify the hierarchical organization of TCM therapeutics, as this directly affects standardization, quality control, and the interpretation of mechanistic data. TCM interventions exist at several distinct levels of complexity: (i) single medicinal herbs (dan wei yao), the raw or processed botanical material from a single species; (ii) processed products (pao zhi pin), in which single herbs undergo standardized processing (e.g., stir-frying, wine-steaming, honey-roasting) that alters chemical composition, bioavailability, and therapeutic properties; (iii) purified extracts, including total flavonoid, total alkaloid, or other chemically defined fractions isolated from single herbs or multi-herb combinations; and (iv) compound formulations (fu fang), classical or modern multi-herb prescriptions in which the therapeutic effect arises from the synergistic interaction of multiple constituents acting on complementary molecular targets. The studies reviewed below span all four levels—from single-herb alkaloid fractions (e.g., motherwort total alkaloids) to multi-herb compound formulations (e.g., Fuke Qianjin Capsule, YiMu-QingGong San)—and readers should note that the level of chemical definition and the strength of mechanistic attribution differ accordingly.

TCM has garnered increasing attention as a complementary therapeutic strategy for endometritis, driven by the limitations of conventional antibiotic monotherapy and the need for mechanistically diverse interventions [94]. In human reproductive medicine, chronic endometritis (CE) is a recognized contributor to recurrent implantation failure (RIF) in IVF-ET. Jin Y et al. (2026) reported a case in which a woman with CE and RIF—who had failed to conceive after four transfers involving seven embryos—achieved spontaneous conception and delivered a healthy baby following integrated TCM–antibiotic therapy, underscoring the potential of integrative approaches to overcome antibiotic-refractory CE [95].

Mechanistically, the NF-κB signaling axis represents the most frequently implicated pathway across TCM formulations studied for endometritis (Table 2). Motherwort total alkaloids (MTAs), comprising 39 alkaloids identified by UPLC-Q-Orbitrap HRMS, attenuated overproduction of inflammatory mediators and promoted endometrial repair in bacteria-induced rat endometritis via the PI3K/AKT/NF-κB cascade [96]. YiMu-QingGong San (YMQGS) suppressed the TLRs/MyD88/TRAF6/NF-κB pathway in LPS-challenged macrophages and in a mouse model of endometritis, concurrently driving M2 macrophage polarization, reducing ROS, and preserving endometrial barrier integrity through upregulation of the tight-junction proteins ZO-1 and occludin [97]. Fuke Qianjin Capsule (FKC) extended this axis to the inflammasome, reversing NLRP3 activation and pyroptosis via TLR4/NF-κB/NLRP3 modulation in both rat tissues and RAW264.7 cells [17]. Similarly, Tiaoqi Jiedu formula downregulated pro-inflammatory cytokines (IL-6, IL-1β, TNF-α, IL-8) while upregulating IL-10 and suppressing TLR4/NF-κB-mediated pyroptosis in a dampness-heat mouse model [98]. Ermiao Fang (EMF), composed of Cortex Phellodendri and Rhizoma Atractylodis, inhibited both NF-κB and MAPK pathways, with binding affinities validated by surface plasmon resonance and pathway suppression confirmed by Western blotting [99].

Beyond NF-κB, several formulations act through distinct signaling axes. Ebing Angong Ye (EBAGY)—composed of Curcuma aromatica and Dryobalanops aromatica—targeted the IL-17 signaling pathway in postpartum sow endometritis (*n* = 80), downregulating IL-17A, TNF-α, IL-6, and IL-1β while restoring vaginal microbiota to healthy-control composition, as demonstrated by 16S rRNA sequencing [100]. Taohong Siwu Decoction (TSD) inhibited ferroptosis through the p38MAPK pathway, shortening uterine bleeding duration in a rat model of incomplete-medical-abortion-induced abnormal uterine bleeding [101]. Salvia miltiorrhiza addressed the crosstalk among inflammation, energy deficiency, and blood stasis, reversing decreased ATP and elevated IL-1β/IL-6/IL-8 in bovine endometrial cells while inhibiting platelet aggregation, with the P38/ERK-AP1 axis identified as the convergent signaling axis [102]. Yimucao formula (YMF) acted on 17 common targets (including BCL2, IL-6, MMP9, and HIF1α) through the AGE-RAGE pathway, improving clinical outcomes in dairy cows [103]. Penning Formula (PNF) identified novel CE-associated targets—Bhmt, Scn10a, and Esr2—via transcriptomic-network pharmacology integration, with molecular docking and RT-qPCR confirming target engagement [26].

An emerging dimension of TCM’s therapeutic action is modulation of the uterine microbiota. Baogong Decoction (BGD) enriched Firmicutes, Lactobacillus, and Lactococcus while depleting Proteobacteria in *E. coli*-induced mouse endometritis. Integrated metabolomics identified dehydroepiandrosterone (DHEA) as a key mediator positively correlated with microbiota restoration, and exogenous DHEA independently ameliorated endometritis [24]. Notably, EBAGY also restored vaginal microbial composition in sows [100], suggesting that microbiota modulation may constitute a shared mechanism across structurally diverse TCM formulations.

An important consideration is whether flavonoids constitute the dominant active class within multi-component TCM formulations or whether their contribution is inseparable from that of co-occurring alkaloids, terpenoids, saponins, and other constituents. Current evidence suggests the latter. In most formulations examined here, network pharmacology analyses identify flavonoids (e.g., quercetin, kaempferol, luteolin, baicalein) among the top-ranked active constituents, but their activity is consistently amplified by non-flavonoid components acting on complementary or overlapping targets. For example, in motherwort total alkaloids the therapeutic effect is primarily driven by 39 alkaloid species rather than flavonoids, whereas in *Salvia miltiorrhiza* the convergent engagement of tanshinones (diterpenoids) and salvianolic acids (phenolic acids) alongside flavonoids produces effects that none achieves alone. This multi-class synergy is precisely what distinguishes TCM from single-compound pharmacology and is arguably its principal therapeutic advantage. From a quality control perspective, individual flavonoid markers (e.g., baicalin content in Scutellaria-based formulas, or total flavonoid content in safflower preparations) are already used in Chinese Pharmacopoeia monographs as authentication and potency indicators. However, given the demonstrated importance of synergistic multi-component activity, single-marker approaches are unlikely to capture the full bioactive profile. Multi-component fingerprinting by UPLC-Q-TOF-MS, coupled with bioactivity-guided fractionation and quantitative contribution analysis, represents a more appropriate quality control paradigm for TCM formulations intended for endometritis management.

Taken together, the current evidence indicates that TCM formulations exert anti-endometritic effects through convergent and complementary mechanisms—predominantly NF-κB suppression, inflammasome inhibition, and pyroptosis attenuation—supplemented by pathway-specific actions on MAPK, IL-17, PI3K/AKT, and ferroptosis signaling, as well as microbiota restoration (Table 2). However, most studies remain preclinical, and rigorously designed randomized controlled trials (RCTs) are warranted to establish clinical efficacy and safety in human patients.

## 6. Current Limitations and How to Resolve Them

**Figure 3:**
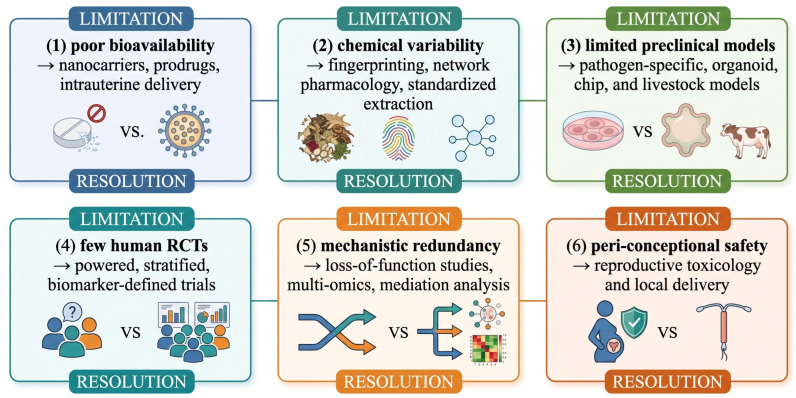
Limitations of flavonoid/TCM therapy for endometritis and their resolutions: (1) poor bioavailability → nanocarriers, prodrugs, intrauterine delivery; (2) chemical variability → fingerprinting, network pharmacology, standardized extraction; (3) limited preclinical models → pathogen-specific, organoid, chip, and livestock models; (4) few human RCTs → powered, stratified, biomarker-defined trials; (5) mechanistic redundancy → loss-of-function studies, multi-omics, mediation analysis; (6) peri-conceptional safety → reproductive toxicology and local delivery.

Notwithstanding the mechanistic richness and translational promise summarized above, six interrelated limitations presently constrain the clinical impact of flavonoid- and TCM-based interventions in endometritis (Figure 3). Each is tractable, and an emerging set of technological and methodological solutions is already reshaping the field.

### 6.1. Poor Pharmacokinetics and Low Oral Bioavailability

The single most consistent barrier to flavonoid translation is unfavorable pharmacokinetics. Aglycones such as luteolin, quercetin, fisetin, apigenin, and naringenin are poorly water-soluble, undergo extensive phase II glucuronidation and sulfation during first-pass intestinal and hepatic metabolism, and reach systemic peak plasma concentrations that are one to two orders of magnitude below those required to achieve the effects demonstrated in vitro [79]. Glycoside conjugation improves intestinal absorption only modestly and frequently masks pharmacological activity until microbial deglycosylation in the distal gut, which is itself variable between individuals. Resolution is now actively pursued along three convergent lines: (i) Nanocarrier delivery—lipid nanoparticles, polymeric micelles, self-nanoemulsifying drug delivery systems (SNEDDS), zein and PLGA nanoparticles and exosome-mimetic vesicles—has been shown to improve oral bioavailability of luteolin, quercetin and EGCG by 5- to 20-fold in animal models and to permit targeted accumulation in inflamed uterine tissue. (ii) Prodrug and co-crystal strategies, including phospholipid complexes (e.g., hesperidin–phytosome) and amino-acid or sugar prodrugs, mask the polyphenolic hydroxyls during absorption and release the parent flavonoid at the target site. (iii) Topical and intrauterine formulations—micronized pessaries, mucoadhesive gels and intrauterine devices loaded with flavonoid-rich extracts such as *Garcinia mangostana–Achyranthes aspera* [92]—bypass first-pass metabolism altogether and deliver high local concentrations with minimal systemic exposure. Combining nanoencapsulation with intrauterine delivery is a particularly promising strategy for gynecological and veterinary contexts where the target tissue is directly accessible.

### 6.2. Chemical Complexity and Batch-to-Batch Variability of Multi-Component Preparations

Multi-herb TCM formulations and crude botanical extracts are by definition mixtures whose composition varies with cultivar, geographic origin, harvest season, processing (pao zhi) and extraction method. This complexity, although increasingly viewed as a strength rather than a weakness in the multi-target paradigm, remains a major regulatory and reproducibility hurdle. Three developments are mitigating this concern. First, comprehensive chemical fingerprinting by UPLC-Q-TOF-MS, UPLC-Q-Orbitrap HRMS and ion mobility–mass spectrometry now permits identification and quantification of dozens of constituents per preparation [18,96]. Second, network pharmacology integrating TCMSP, SwissTargetPrediction and STRING databases—exemplified by recent analyses of YiMu-QingGong San, Yimucao formula and Penning Formula—deconvolutes complex preparations into a small set of core active constituents and target proteins amenable to standardization [26,97,103]. Third, green and reproducible extraction technologies such as deep-eutectic-solvent ultrasound-assisted extraction (DES-UAE) of Syringa oblata flavonoids [64] provide a chemometric basis for quality-by-design specifications. Together, these approaches make it feasible to define a chemically defined, bioactive marker-anchored standard for individual TCM preparations.

### 6.3. Over-Reliance on a Narrow Set of Preclinical Models

The current evidence base is dominated by acute lipopolysaccharide-induced murine endometritis and immortalized endometrial epithelial cell lines (HEEC, Ishikawa, bEEC, BEND). These models faithfully recapitulate the early TLR4/NF-κB/NLRP3 axis but capture only a fraction of the chronic, polymicrobial, microbiota-dependent and immune-tolerant biology of human chronic endometritis and bovine subclinical disease. Pathogen-specific models (*Staphylococcus aureus*, *E. coli*, *Trueperella pyogenes*, *Leptospira*) and bacteria-induced rat models [96], are more representative but remain underutilized, while organoid and microfluidic endometrial-on-chip platforms—capable of incorporating epithelial, stromal, immune and microbial compartments simultaneously—are only beginning to be applied to flavonoid and TCM screening. A deliberate diversification toward (i) pathogen-specific and polymicrobial in vivo models, (ii) chronic and recurrent disease paradigms, (iii) human endometrial organoids derived from CE patients, and (iv) naturally diseased livestock as preclinical-to-clinical bridges should be prioritized in future preclinical work.

A related limitation is the insufficient attention to disease heterogeneity. Human chronic endometritis and bovine postpartum endometritis differ markedly in etiology, dominant pathogen spectrum, immune microenvironment, and tissue architecture, yet the mechanistic conclusions derived from one species are frequently extrapolated to the other without qualification. Within human medicine alone, CE associated with Enterococcus-dominant infections may differ in its inflammatory profile and treatment response from CE driven by Mycoplasma or Chlamydia, and endometritis in the context of intrauterine devices, post-cesarean infection, or pelvic inflammatory disease each presents distinct host-pathogen dynamics. Likewise, in veterinary medicine, the distinction between clinical, subclinical, and cytological endometritis is not merely semantic but reflects different degrees of immune activation and may predict differential responsiveness to anti-inflammatory flavonoid therapy. Future studies should explicitly define the disease subtype under investigation, use diagnostic criteria appropriate to the subtype (e.g., CD138 immunohistochemistry for human CE, endometrial cytology thresholds for bovine subclinical endometritis), and avoid generalizing conclusions across subtypes without supporting evidence.

### 6.4. Scarcity of Adequately Powered RCTs in Humans

With the exception of MPFF in cattle [89] and isolated case reports of integrative TCM–antibiotic therapy in CE [95], the human clinical literature on flavonoids and TCM in endometritis remains dominated by small, single-center, often unblinded studies. Translation requires three concrete advances. First, well-powered, multicenter, double-blind, placebo- or active-comparator-controlled trials with histologically and microbiologically defined entry criteria (e.g., CD138-positive plasma cells per high-power field; 16S rRNA-defined dysbiosis). Second, harmonized outcome measures spanning histological cure, microbiota restoration, implantation rate, and live birth rate in human trials, and cure rate, days open, and pregnancy per AI in livestock. Third, biomarker-stratified designs in which patients or animals are selected for trials based on the molecular axis presumed to be engaged—Nrf2 signature, NLRP3 activation, microbiota composition—rather than on syndromic diagnosis alone. The convergence of digital pathology, single-cell transcriptomics of endometrial biopsies and metagenomic sequencing of the reproductive-tract microbiome makes such stratification feasible within the next trial cycle.

### 6.5. Mechanistic Redundancy and the Challenge of Attributing Efficacy

The multi-target nature of flavonoids and TCM is therapeutically attractive but analytically challenging: when a compound simultaneously inhibits TLR4/NF-κB, activates Nrf2, suppresses NLRP3, and remodels the microbiota, attributing efficacy to a single mechanism is often impossible. This is a methodological rather than a conceptual problem and is being addressed by combining genetic and pharmacological loss-of-function approaches (Nrf2-knockout mice, NLRP3-deficient lines, AMPK and SIRT1 inhibitors compound C and EX-527 [74]; PPAR-γ siRNA and GW9662 [85,86] with quantitative multi-omics). Causal mediation analysis applied to paired tissue and microbiome data—as exemplified by the hyperoside–HPLA–TLR4 axis [94]—provides a statistical framework for partitioning effect size across mechanisms and identifying the minimum sufficient set of targets for efficacy.

### 6.6. Safety in the Peri-Conceptional and Pregnant Population

Among the flavonoids reviewed here, those with the highest estrogenic risk are the isoflavones (genistein, daidzein) and certain flavones. Apigenin binds ERβ with an IC50 in the low micromolar range and has been shown to modulate ERα-dependent transcription in endometrial Ishikawa cells at concentrations achievable by supplementation. Luteolin similarly engages both ERα and ERβ and can stimulate estrogen-responsive gene expression at pharmacological doses, raising the concern that it may interfere with the tightly regulated endometrial decidualization program during the implantation window. Naringenin, although weaker, has demonstrated ERα agonist activity in reporter gene assays and should not be assumed inert. Puerarin (from Pueraria lobata) is structurally related to daidzein and possesses documented phytoestrogenic activity, while kaempferol and quercetin display weak but measurable ER binding. The clinical significance of these interactions at the doses proposed for endometritis therapy remains unknown, and the risk is compounded for TCM compound formulations in which multiple phytoestrogenic flavonoids may be co-administered. Dedicated in vitro endometrial decidualization assays and in vivo implantation-window exposure studies are essential before any flavonoid-based intervention can be recommended in women undergoing assisted reproduction.

Because endometritis is most consequential in women actively pursuing pregnancy, any therapeutic agent must demonstrate an exceptional safety profile in the peri-implantation window and, ideally, in early gestation. Flavonoids are widely consumed in the human diet, but pharmacological doses—particularly of phytoestrogenic flavones such as apigenin and luteolin and of isoflavones—can engage estrogen receptors and modulate the decidualization and implantation programs. Likewise, several TCM herbs (e.g., Carthamus tinctorius, used in safflower total flavonoids) have traditional contraindications in pregnancy. Resolution requires (i) systematic reproductive toxicology testing of lead compounds in OECD-compliant fertility and embryo–fetal development studies, (ii) explicit dose–response evaluation across the peri-implantation window, and (iii) preferential development of intrauterine and locally delivered formulations that minimize systemic and embryo–fetal exposure. Until such data are available, peri-conceptional use should be confined to controlled trials.

### 6.7. Herb–Drug Interactions and Polypharmacy Risks

A critical yet underexplored dimension of the adjunctive strategy advocated here is the potential for clinically significant pharmacokinetic and pharmacodynamic herb–drug interactions when flavonoids or TCM formulations are co-administered with conventional antibiotics. These constituents are orally bioavailable small molecules that engage the same enzymes and transporters governing antibiotic absorption, distribution, metabolism and elimination, and the nanocarrier delivery proposed in Section 6.1 can only raise the systemic concentrations at which such interactions occur. The best-characterized mechanism is reversible inhibition of intestinal and hepatic cytochrome P450 (CYP) enzymes: quercetin, kaempferol and apigenin inhibit CYP3A4, CYP1A2 and CYP2C9 at low-to-mid micromolar concentrations attainable in vivo [104,105,106]. Because these same flavonoids are repeatedly nominated as principal active constituents of the formulations reviewed here—quercetin, kaempferol and apigenin in Yimucao formula [103], and quercetin and kaempferol across Fuke Qianjin Capsule and YiMu-QingGong San [17,97]—this risk is intrinsic to the candidate preparations rather than hypothetical. CYP3A4 inhibition can prolong exposure to macrolides (azithromycin, erythromycin) used in *Mycoplasma genitalium* and bovine uterine infection, compounding azithromycin’s recognized QT-prolongation liability [107], while CYP1A2 and CYP2C9 inhibition can comparably elevate fluoroquinolone and metronidazole exposure; conversely, CYP1A2 induction by chrysin and genistein could drive subtherapeutic fluoroquinolone levels that favor resistance selection.

A second tier involves drug transporters [108,109]. Baicalin and its aglycone baicalein—the principal flavonoids of the Scutellaria-based preparations reviewed in Section 4.7 [88]—inhibit OATP1B1 and P-glycoprotein at clinically attainable concentrations [106,110,111], raising systemic exposure to substrates such as rifampicin and doxycycline, the latter a first-line agent for *Chlamydia trachomatis*–associated chronic endometritis. Glycyrrhizin, which co-occurs with the flavonoid liquiritin in the Glycyrrhiza-based formulations discussed in Section 4.4 [75], inhibits 11β-hydroxysteroid dehydrogenase type 2 and may precipitate pseudohyperaldosteronism when combined with the glucocorticoid support frequently given to women with recurrent implantation failure [112,113]—the precise population for whom TCM adjuncts are proposed [95]. Such patients concurrently receive progesterone, gonadotrophins, low-dose aspirin, heparin and, in some protocols, tacrolimus: flavonoid antiplatelet activity is additive with aspirin, and CYP3A4/P-gp inhibition can unpredictably elevate the narrow-therapeutic-index substrate tacrolimus. That over-the-counter flavonoid supplements are seldom disclosed to prescribers turns this into a silent polypharmacy risk that current pharmacovigilance is not designed to detect.

In food-producing animals the dominant concern shifts from systemic toxicity to drug residues. Withdrawal periods and maximum residue limits under EU Regulation 2019/6—already a binding constraint on veterinary antibiotic use noted in Section 3—are derived from single-agent pharmacokinetics, so flavonoid-mediated CYP or P-gp inhibition that slows elimination of oxytetracycline, ampicillin or cephalosporins could invalidate established withdrawal intervals and generate residue violations undetectable by current monitoring. Multi-herb formulations magnify every preceding concern, since flavonoids, alkaloids, terpenoids and saponins act together on overlapping enzymes and transporters; network pharmacology and in silico ADME tools (SwissADME, pkCSM, the FDA interaction database) provide a tractable preclinical screen that should precede any combination-antibiotic trial [114]. Not all interactions are adverse: P-gp inhibition by baicalein might enhance intracellular antibiotic penetration into the macrophage-resident *C. trachomatis* and *Trueperella pyogenes* described in Section 3, converting an apparent liability into a therapeutic mechanism—a possibility resolvable only by studies measuring pharmacokinetic and microbiological endpoints in parallel.

No preclinical study reviewed here assessed herb–antibiotic pharmacokinetics, and no clinical or veterinary report controlled for concomitant medication, leaving the safety of the very combination proposed uncharacterized in every species. Pending formal interaction studies, three interim measures are warranted: prescribers and veterinarians should be advised that flavonoid and TCM products modulate CYP and transporter activity, so that co-administration with narrow-therapeutic-index antibiotics—macrolides, tetracyclines and fluoroquinolones—warrants caution and, where feasible, therapeutic drug monitoring; trials of flavonoid–antibiotic combinations should incorporate mandatory pharmacokinetic sub-studies sampling peak and trough antibiotic concentrations; and pharmacovigilance systems in both human and veterinary medicine should record concurrent herbal and TCM use as a structured data field, enabling post-market detection of interactions currently invisible to surveillance.

## 7. Conclusions

Endometritis exemplifies a class of inflammatory reproductive disorders for which antibiotic monotherapy is necessary but no longer sufficient. Antimicrobial resistance, microbiota disruption, tissue residues and persistent post-cure infertility have together redefined it as a host-driven inflammatory syndrome whose management demands interventions that simultaneously suppress maladaptive innate immunity, restore redox and barrier integrity, and re-establish a eubiotic mucosal ecosystem. The evidence synthesized here positions flavonoids and traditional Chinese medicine as mechanistically plausible candidates for this role, pending validation in adequately powered clinical trials, converging on a tractable set of nodes—TLR4/NF-κB, NLRP3-driven pyroptosis, Nrf2/HO-1 redox defense, PI3K/AKT and PPAR-γ signaling, ferroptosis and the gut–uterus axis—that single-mechanism antibiotics inherently lack.

Veterinary field studies and integrative human case reports show that this promise has shown early signals of clinical benefit in limited studies, particularly where these agents act as host-directed adjuncts that convert antibiotic non-responders into responders. The principal remaining barriers—pharmacokinetics, formulation heterogeneity, narrow preclinical models, scarce randomized trials, attribution of efficacy and peri-conceptional safety—are individually well-defined and collectively tractable through nanocarrier and intrauterine delivery, network-pharmacology-guided standardization, organoid and naturally diseased livestock models, and biomarker-stratified trials. In an environment that increasingly demands a One Health approach to antimicrobial stewardship, the value of flavonoids and TCM lies not in displacing antibiotics but in reducing their use, restoring their effectiveness where they are failing, and resolving the inflammatory and dysbiotic legacy that antibiotics alone cannot address.

A SWOT (Strengths, Weaknesses, Opportunities, Threats) framework usefully consolidates the strategic position of flavonoid and TCM-based interventions in endometritis. *Strengths* include the multi-target pharmacology simultaneously addressing inflammation, oxidative stress, regulated cell death, and microbiota dysbiosis; the extensive empirical and ethnopharmacological precedent from centuries of TCM use; the growing body of mechanistic evidence supported by validated loss-of-function approaches; and the demonstrated veterinary field efficacy in naturally affected livestock. *Weaknesses* encompass poor oral bioavailability and unfavorable pharmacokinetics of most aglycone flavonoids; batch-to-batch variability of multi-component TCM preparations; the near-exclusive reliance on acute, LPS-only rodent models; the scarcity of adequately powered randomized controlled trials in humans; publication bias toward positive results; the difficulty of attributing therapeutic efficacy to individual mechanisms within multi-target agents; and the limited reproductive toxicology data, particularly for phytoestrogenic compounds in the peri-implantation window. Opportunities are represented by the rapid advancement of nanocarrier and intrauterine delivery technologies that can overcome bioavailability barriers; the maturation of network pharmacology, UPLC-Q-TOF-MS fingerprinting, and bioactivity-guided fractionation for standardization; the availability of human endometrial organoids and organ-on-chip platforms for higher-fidelity preclinical testing; the convergence of digital pathology, single-cell transcriptomics, and metagenomic sequencing enabling biomarker-stratified trial designs; and the increasingly favorable regulatory environment for integrative and adjunctive therapies within One Health antimicrobial stewardship frameworks. Threats include the potential for pharmacokinetic herb–drug interactions with co-administered antibiotics; the risk of regulatory fragmentation across different national pharmacopoeias and food-supplement versus drug classification systems; the possibility that multi-component complexity will prove an irreducible barrier to regulatory approval under conventional single-compound paradigms; and the danger that premature clinical adoption without adequate safety data could discredit the field and delay legitimate translation. Addressing the weaknesses and mitigating the threats through the roadmap proposed above—standardized preparations, diversified preclinical models, herb–drug interaction studies, reproductive toxicology testing, and biomarker-stratified trials—is the most direct path from mechanistic promise to clinical impact.

## Figures and Tables

**Table 1:** Flavonoids and flavonoid-rich preparations with reported activity against endometritis.

Flavonoid	Botanical/Dietary Source	Principal Target Pathway	Model System	Reference
Luteolin	Syringa oblata, dietary	TLR4/NF-κB and Nrf2/HO-1; suppresses TNF-α, IL-1β, IL-6, MPO, MDA and ROS, attenuates ferroptosis and restores ZO-1/occludin barrier integrity	Mouse; in vitro	[21,22,64,79,80]
Hyperoside	Hawthorn (Crataegus)	Gut–uterus axis; enriches *Lactobacillus*/*Prevotella*, raises HPLA that binds TLR4 and inhibits TLR4/NF-κB; also represses TGF-β/Smad3 fibrosis	Mouse; rat	[23,87]
Liquiritin	*Glycyrrhiza glabra*	Keap1/Nrf2/HO-1; lowers ROS, raises SOD/CAT and suppresses IL-1β, TNF-α, IL-6 (abolished by ML385)	HEECs	[75]
Fisetin	Fruits, vegetables	TLR4–ROS/NF-κB inhibition with Nrf2/HO-1 activation; also reduces lesion size, MC-derived NLRP3 and TGF-β in endometriosis	Mouse; BEND; rat	[77,78]
Engeletin	White grapes, wine	TLR4/MyD88/IRAK1/TRAF6/TAK1–NF-κB; suppresses iNOS, COX-2 and pro-inflammatory cytokines	Mouse	[19]
Hesperidin	Citrus	AMPK/PGC-1α activation (direct AMPK binding); restores mitochondrial function and lowers ROS, MPO, CD38/CD138	HEECs; mouse	[20]
Cyanidin-3-O-glucoside	Anthocyanin-rich plants	NF-κB suppression with PPARγ/ABCA1 activation (reversed by GW9662); anti-oxidative	Mouse	[86]
Astilbin	*Smilax china*	PPAR-γ–dependent inhibition of TLR4/IL-6R–MyD88–NF-κB and JAK2/STAT3 positive feedback	Rat; EECs	[85]
Morin	Fruits, dietary	TLR4/NF-κB inhibition and Nrf2 activation; curbs inflammation, oxidative stress and ferroptosis (MDA, iron, GSH, ATP)	Mouse	[81]
Puerarin	*Pueraria*	AMPK/SIRT1/GPX4 and P2X7/NLRP3 regulation with NF-κB suppression; inhibits ferroptosis	Mouse; MEECs	[73,74]
Naringin	Citrus, dietary	Inhibits the ERS–PI3K/AKT axis, reducing UPR-driven autophagy and apoptosis	Mouse; bEEC	[84]
Icariin	*Epimedium brevicornum*	TLR4-associated NF-κB suppression and Nrf2 activation (NQO1, HO-1, GCLC); restores IL-10 and antioxidant enzymes	Mouse; MEECs	[70]
Apigenin	Dietary flavone	NF-κB/Nrf2/HO-1; inhibits MPO, NF-κB, TNF-α and IL-1β, raises Nrf2/HO-1 and lowers MDA	MEECs	[76]
EGCG	Green tea (Camellia sinensis)	SIRT1 upregulation with NLRP3/ASC/Caspase-1 inhibition; lowers oxidative stress and apoptosis	Mouse; BEECs	[72]
Baicalin	*Scutellaria*	NF-κB and JNK suppression (p65, IκBα, JNK phosphorylation); lowers iNOS, IL-1β, TNF-α	Rabbit; RAW264.7	[88]
Astragalin	Multiple TCM herbs; *Urtica dioica*	TLR4/NF-κB and MAPK (p38, ERK, JNK) suppression; modulates JAK/STAT	Mouse; SEECs; EECs	[66,67,68,69]
Safflower total flavonoids	*Carthamus tinctorius*	ERα/PI3K/AKT activation with ASK1/JNK/caspase suppression; restores gut microbiota balance	Ishikawa; rat	[82,83]
TFC (Clinopodium chinense)	*Clinopodium chinense*	TLR4/NF-κB/NLRP3 suppression; inhibits pyroptosis (caspase-1, GSDMD, ASC)	Mouse; MEECs	[18]
MPFF	Citrus (diosmin/hesperidin)	Clinical anti-inflammatory and antibacterial action; improves uterine involution, PMN profile and fertility	Dairy cows	[89]

Abbreviations: BEECs/BEND/bEEC, bovine endometrial epithelial cells; EECs, endometrial epithelial cells; EGCG, epigallocatechin-3-gallate; HEECs, human endometrial epithelial/endothelial cells; HPLA, hydroxyphenyllactic acid; LPS, lipopolysaccharide; MEECs, mouse endometrial epithelial cells; MPFF, Micronised Purified Flavonoid Fraction; MPO, myeloperoxidase; SEECs, sheep endometrial epithelial cells; TFC, total flavonoids of *Clinopodium chinense*; UPR, unfolded protein response.

**Table 2:** Summary of TCM formulations investigated for the treatment of endometritis.

Formulation	Active Constituents/Source	Principal Target Pathway and Effect	Model System	Reference
FKC	5 active components (HPLC)	TLR4/NF-κB/NLRP3; lowers MPO, IL-18, IL-1β, IL-6, TNF-α, NLRP3, Caspase-1 and GSDMD; inhibits inflammasome activation and pyroptosis	LPS-induced rat model; LPS-induced RAW264.7	[17]
BGD	DHEA, catechol (metabolomics)	Microbiota–metabolite axis; raises Firmicutes, Lactobacillus and DHEA and lowers Proteobacteria; restores uterine microbiota and reduces inflammation	*E. coli*-induced mouse endometritis	[24]
PNF	25 active ingredients (database mining)	Stress response/prolactin signaling (Bhmt, Scn10a, Esr2); lowers Bhmt, Scn10a and Esr2 expression; identifies novel CE therapeutic targets	Rat CE model (transcriptomics)	[26]
MTAs	39 alkaloids (UPLC-Q-Orbitrap HRMS)	PI3K/AKT/NF-κB; lowers inflammatory mediators and promotes endometrial cell repair; reduces inflammation and promotes tissue repair	Bacteria-induced rat endometritis; LPS-stimulated RAW 264.7 and HEECs	[96]
YMQGS	Components identified (UHPLC/MS)	TLRs/MyD88/TRAF6/NF-κB; lowers TNF-α, IL-6, IL-1β and ROS, drives M2 polarization and raises ZO-1/occludin; protects endometrial barrier and reduces fibrosis	LPS-stimulated RAW264.7; LPS-induced mouse model (mouse/bovine)	[97]
Tiaoqi Jiedu	Not specified	TLR4/NF-κB; lowers IL-6, IL-1β, TNF-α, IL-8, NLRP3, GSDMD and caspase-1 and raises IL-10; anti-inflammatory and anti-pyroptosis effects	Mouse endometritis (dampness-heat)	[98]
EMF	24 compounds (UHPLC-Q-TOF/MS); 8 absorbed	NF-κB and MAPK; inhibits NF-κB/MAPK pathway proteins; reduces uterine inflammation	Rat endometritis model	[99]
EBAGY	Borneol, curzerene, curdione, germacrone (GC-MS)	IL-17 signaling; lowers IL-17A, TNF-α, IL-6 and IL-1β; restores vaginal microbiota and improves reproductive performance	Postpartum sows; PEECs	[100]
TSD	Classic multi-herb formula	p38 MAPK/ferroptosis; lowers ferroptosis markers and inflammatory factors; shortens bleeding duration and decreases bleeding volume	IMA-induced AUB rat model; Ishikawa cells	[101]
Salvia miltiorrhiza	Tanshinone IIA, cryptotanshinone, salvianolic acid A/B	TLR and platelet-activation signaling with AMPK (P38/ERK–AP1 crosstalk); lowers IL-1β, IL-6 and IL-8, raises ATP and reduces platelet aggregation; resolves inflammation–energy–stasis crosstalk	Dairy cows; bovine endometrial cells; blood-stasis rat model	[102]
YMF	Quercetin, kaempferol, β-sitosterol, apigenin, isorhamnetin (TCMSP)	AGE-RAGE and fluid shear stress; acts on 17 common targets (BCL2, IL-6, MMP9, HIF1α, TNF, IL-1β, ICAM1); improves clinical symptoms	Lactation cows with endometritis (bovine)	[103]

Abbreviations: AUB, abnormal uterine bleeding; CE, chronic endometritis; DHEA, dehydroepiandrosterone; EBAGY, Ebing Angong Ye; EMF, Ermiao Fang; FKC, Fuke Qianjin Capsule; IMA, incomplete medical abortion; LPS, lipopolysaccharide; MTAs, motherwort total alkaloids; PNF, Penning Formula; RIF, recurrent implantation failure; SPR, surface plasmon resonance; TCM, traditional Chinese medicine; TSD, Taohong Siwu Decoction; WB, Western blotting; YMF, Yimucao formula; YMQGS, YiMu-QingGong San; BGD, Baogong Decoction; ROS, reactive oxygen species.

## Data Availability

No new data were created or analyzed in this study.
